# 3D biomimetic artificial bone scaffolds with dual-cytokines spatiotemporal delivery for large weight-bearing bone defect repair

**DOI:** 10.1038/s41598-017-08412-0

**Published:** 2017-08-10

**Authors:** Xiaogang Bao, Lingjun Zhu, Xiaodong Huang, Dezhi Tang, Dannong He, Jiangang Shi, Guohua Xu

**Affiliations:** 1 Department of Orthopedic Surgery, Changzheng Hospital, Naval Medical University, 415 Fengyang Road, Shanghai, 200003 People’s Republic of China; 2 Longhua Hospital, Shanghai University of Traditional Chinese Medicine, 725 South Wanping Road, Shanghai, 200032 People’s Republic of China; 3 National Engineering Research Center for Nanotechnology, Shanghai, 200241 People’s Republic of China

## Abstract

It is a great challenge to prepare “functional artificial bone” for the repair of large segmental defect, especially in weight-bearing bones. In this study, bioactive HA/PCL composite scaffolds that possess anatomical structure as autogenous bone were fabricated by CT-guided fused deposition modeling technique. The scaffolds can provide mechanical support and possess osteoconduction property. Then the VEGF-165/BMP-2 loaded hydrogel was filled into biomimetic artificial bone spatially to introduce osteoinduction and angioinduction ability via sustained release of these cytokines. It has been revealed that the cytokine-loaded hydrogel possessed good biodegradability and could release the VEGF-165/BMP-2 sustainedly and steadily. The synergistic effect of these two cytokines showed significant stimulation on the osteogenic gene expresssion of osteoblast *in vitro* and ectopic ossification *in vivo*. The scaffolds were then implanted into the rabbit tibial defect sites (1.2 cm) for bone regeneration for 12 weeks, indicating the best repair of defect *in vivo*, which was superior to the pure hydrogel/scaffolds or one-cytokine loaded hydrogel/scaffolds and close to autogenous bone graft. The strategy to construct an “anatomy-structure-function” trinity system as functional artificial bone shows great potential in replacing autogenous bone graft and applying in large bone defect repair clinically in future.

## Introduction

More than 15 million people suffered from bone fracture or defect caused by accidents or diseases all over the world each year^1^, of which 10% showed complication as bone ununion that resulted from the subsequently unsuccessful recovery or repair^2^. It is highly possible that the lack of integration between host bone and the substitution lead to non-unions with late graft facture^3^. The golden standard of bone repair, autogenous bone graft, has disadvantages such as limited source and extensive surgery, let along the possible rejection reaction and viral transmission caused by the second choice, allograft bone^4^. Under this circumstance, proper bone tissue engineering scaffolds, especially for the large bone defect repair, which could mimic the structure as well as the function of autogenous bone are urgently needed.

One of the most important factor of biomimitic bone scaffold is the structure and mechanical property^5^. The hierarchical structure (cortical bone/cancellous bone/medullary canal) should be fabricated with bone-like components. It is noticed that the 3D printing technique, which has become a hot spot in bioengineering scaffolds preparation, could effectively control the porous structure via layer-by-layer deposition according to the computer-aided design model^6^. On the other hand, the natural bone is composed of inorganic hydroxyapatite and organic collagen fibers, indicating that a similar composite material should be considered as a replacement^7^. Polymer possessing favorable degradability and satisfactory biocompatibility such as collagen, polycaprolactone and chitosan has been applied to combine with hydroxyapatite or other bioceramic particles and showed good osteoinduction property *in vitro* and *in vivo*, which offered multiple choice for 3D printing technique^8^.

Another key factor of biomimitic bone is the ability to reconstruct blood vessels and to support the new bone formation. Deficiencies in vascularity could lead to cell apoptosis, undesirable bone growth^9^ and a further delayed bone union^3^. While traditional approaches including vascular pedicle and bundel bone grafting had limited application and high requirements on microsurgery^10^, the strategies by using proper growth factors have attracted much attention in recent years. They can target specific cellular receptors and animatedly trigger various cellular processes^11, 12^. Specifically, BMP, which is essential in osteoinduction^13, 14^, among which BMP-2 and BMP-7 have been approved by FDA to apply in clinic^15^, and VEGF that could modulate angiogenesis^16^, have been proved to interact with each other during bone formation^8, 17^. While the sustained release of these growth factors is critical for their efficaciy, it is important to optimize the spatiotemporal delivery of BMP and/or VEGF under lower concentration by choosing appropriate carrier materials^18^. It has be revealed that injectable thermosensitive hydrogels were a promising candidate^19, 20^. Such a hydrogel system was a free-flowing polymer solution at room temperature and spontaneously turned into a semi-solid gel at body temperature. Therefore, fragile therapeutic agents, like protein or other small molecules, were conveniently entrapped by simply mixing them and then the drug-loaded polymer solution was introduced into the body by injection, followed by the formation of *in situ* hydrogel at the target site as a sustained drug release depot. Injectable thermosensitive PLGA-PEG-PLGA hydrogels are a very attractive system for delivery of various drugs due to the easy preparation, adjustable biodegradability and good biocompatibility^21^.

In this study, a biomimitic HA/PCL scaffold was prepared by 3D printing technique and a thermosensitive PLGA-PEG-PLGA hydrogel containing BMP/VEGF was then loaded into the porous scaffolds. We used the obtained scaffold as biomimic bone and systematically studied the osteogensis property *in vitro* and *in vivo*. It is expected that the composite scaffold could be served as an “anatomy-structure-function” trinity system in weight-bearing defect repair (Figure 13).

## Results

### Fabrication and characterization of artificial bone scaffolds

**Figure 1:**
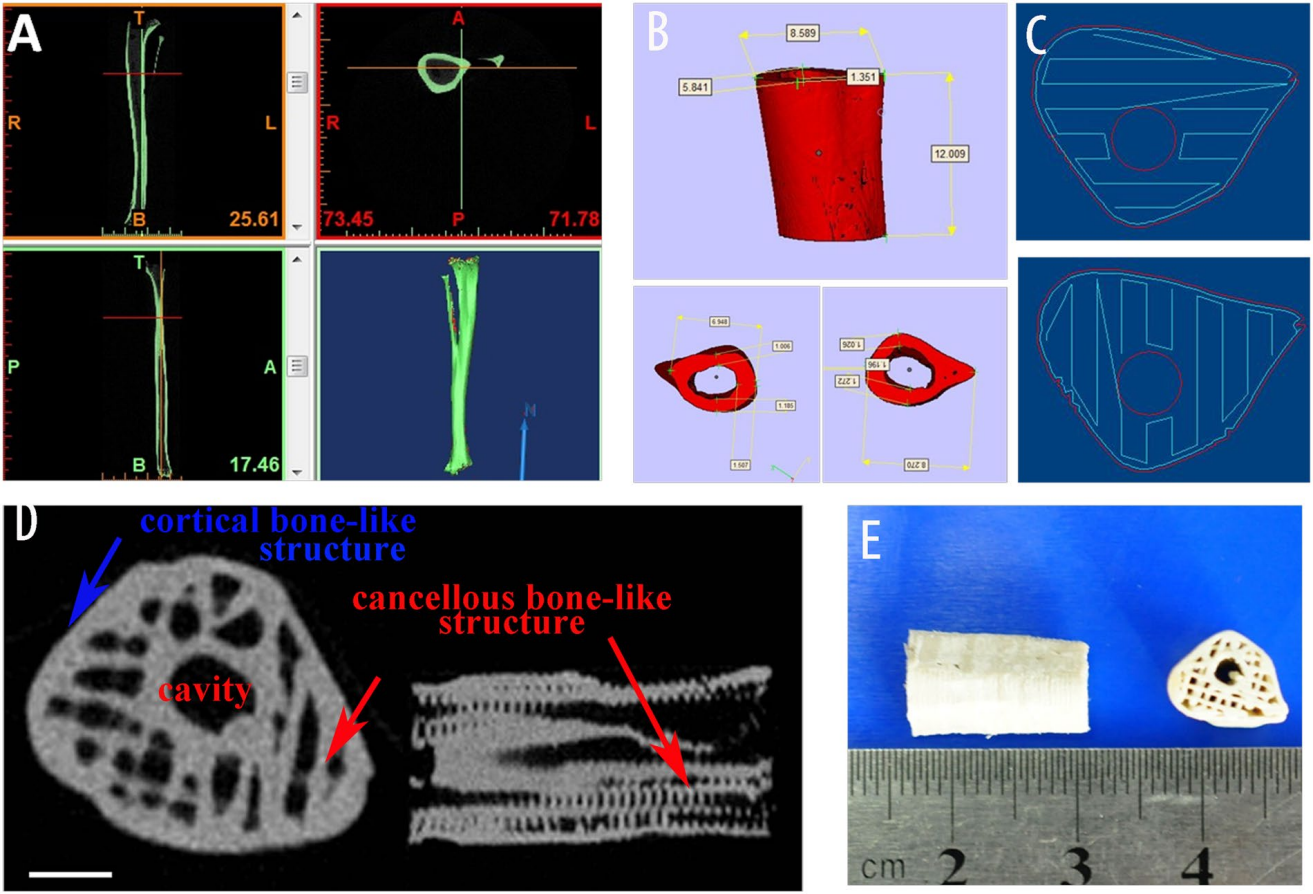
(**A**) CT scanning of rabbit leg, (**B**) reconstruction of 1.2 cm-long tibia structure, (**C**) the fabrication path of FDM machine, (**D**) CT scanning of prepared artificial bone scaffolds, (**E**) The photograph of artificial bone scaffolds.

The morphology and structure of rabbit tibia was precisely presented via CT scanning and a 1.2 cm-long tibia was selected and reconstructed (Fig. 1A,B). Melt HA/PCL paste was fabricated layer by layer according to the fixed path (Fig. 1C) with the FDM technique. A biomimetic dense cortical bone outside the scaffolds and the interconnected porous structure (size of hundreds of micrometers) of the bone scaffold was clearly shown by CT scanning and photograph (Fig. 1D,E). The scaffolds possessed a compressive strength of 20.65 MPa, which was 8 times higher than that of commercial BAM scaffolds although only the quarter of that of autogenous bone. Also, the tensile modulus of the scaffolds, valued at 78.33 Mpa, is more than twice of BAM scaffolds. The compressive strength and elastic modulus of artificial and natural bones were shown in Table 1.

**Table 1:** Mechanical properties of 3D printed scaffolds.

sample	Compressive Strength (MPa)	Tensile Modulus (MPa)
Artifical Scaffold	20.65 ± 1.64	78.33 ± 2.82
BAM Scaffold	2.49 ± 0.48	32.03 ± 8.45
Autogenous bone	95.94 ± 6.93	2.043 ± 0.20 *10^4

### The characterization of PLGA-PEG-PLGA hydrogel

**Figure 2:**
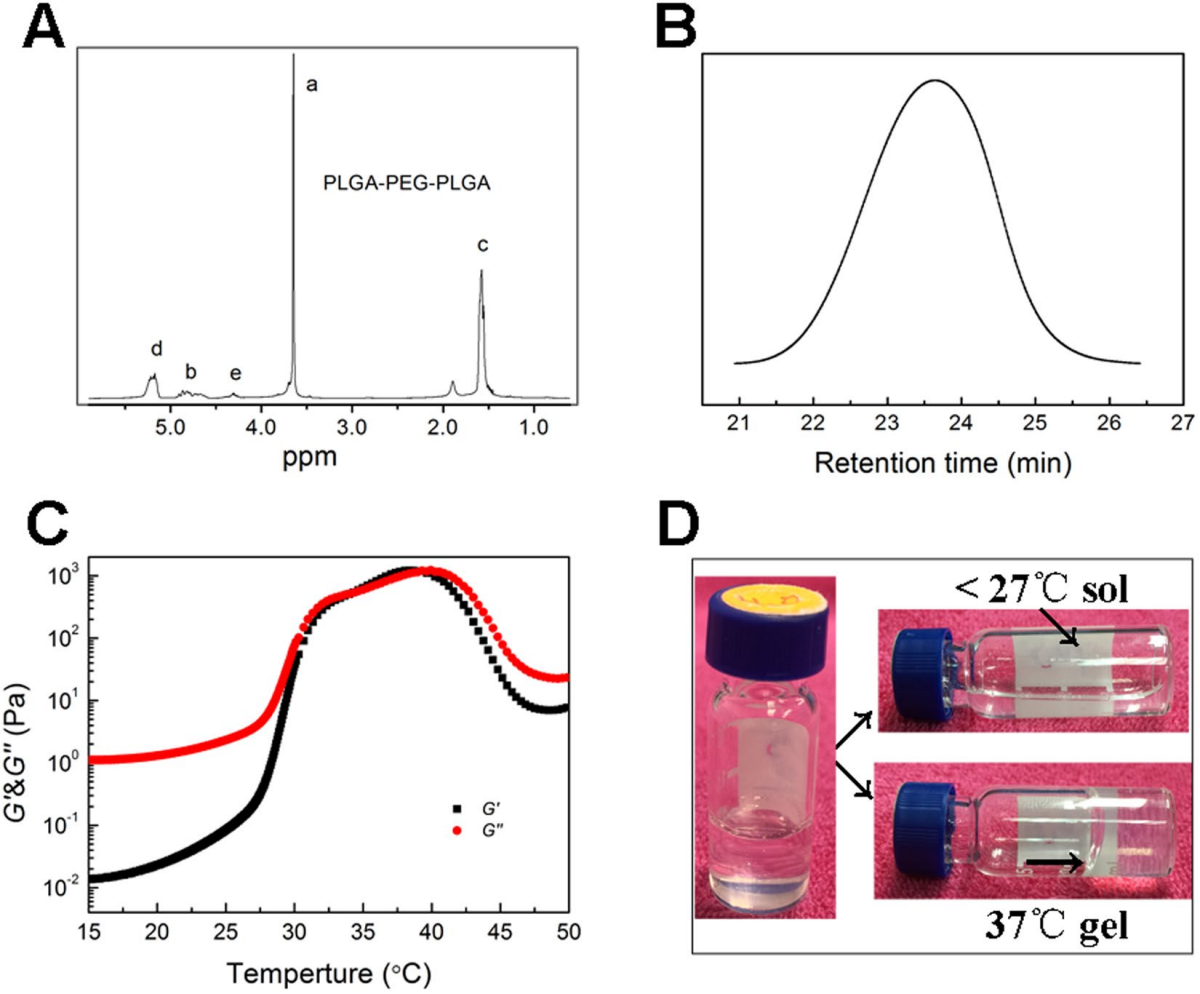
(**A**) ^1^H NMR specturm of the PLGA-PEG-PLGA copolymer, (**B**). GPC analysis of the copolymer shows a unimodal distribution, (**C**) the sol-gel transition process of the copolymer-saline solution (transition temperature of 35 °C), (**D**) the apperance of copolymer-saline solution at different temperatures.

As revealed by the ^1^H NMR analysis, the synthesized copolymer showed a ratio of LA and GA at 4:1 with the number-average molecualr weight (*M*_n_) of 1740-1500-1740 (Fig. 2A). GPC test further proved the copolymer exhibited a unimodal distribution with polydispersity of 1.26 (Fig. 2B).

The dynamic rheological measurement was conducted to analyse the sol-gel transition of PLGA-PEG-PLGA copolymer (dissolved in 25 wt% saline). As indicated in Fig. 2C, the storage modulus G′ showed the load-bearing capacity while the loss modulus G″ gave the energy dissipating capacity during a cyclic deformation. At low temperature under 27 °C, the value of G″ is higher than G′, thus the coploymer solution was a free flowing liquid. With the increasing of temperature (27–35 °C, step of 0.5 °C/min), both the G″ and G′ abruptly increased. The sol lost its flowability due to high viscosity and turned into a semi-solid gel (Fig. 2D). The value of G″ and G′ equaled at 35 °C, which is also the named as the sol-gel transition temperature. The system was suitable for injection as hydrogel by then. The amphiphilic PLGA-PEG-PLGA copolymers tend to form micelles to reduce free energy, and the micellar aggregation and formation a percolated micellar network upon heating driven by hydrophobic interaction was responsible the sol-gel transition of such a hydrogel^22, 23^.

### The growth factors loading and release behavior in PLGA-PEG-PLGA hydrogel

**Figure 3:**
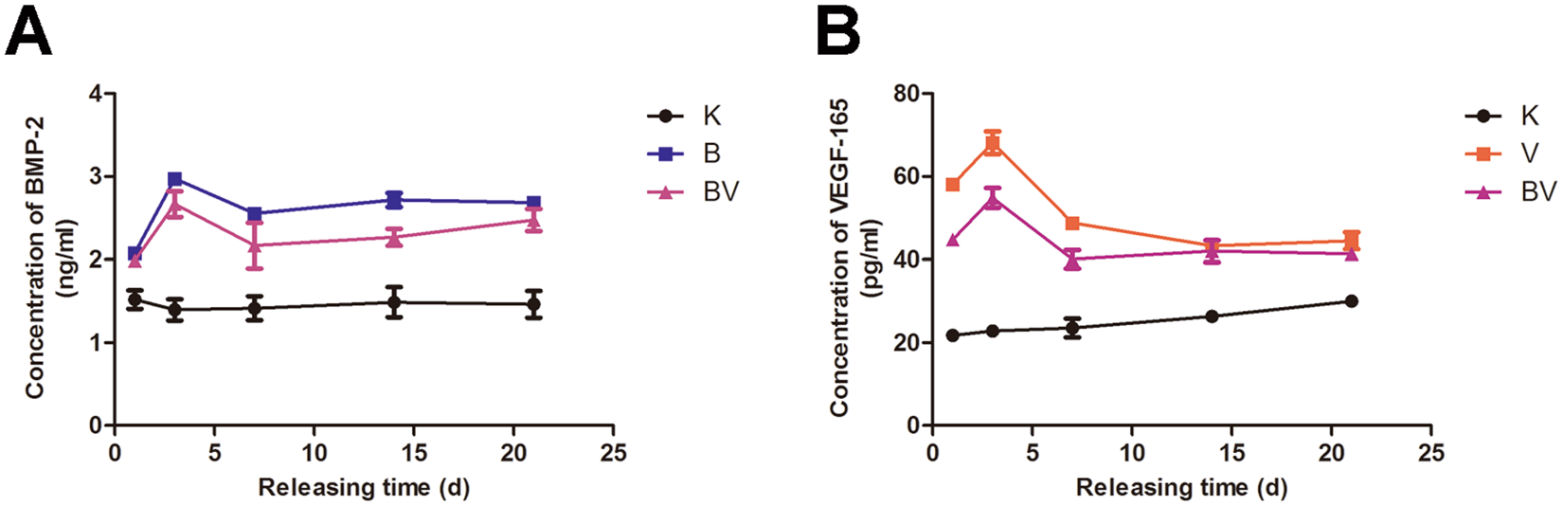
(**A**) BMP-2 release in PBS of different groups for 21 days, (**B**). VEGF-165 release in PBS of different groups for 21 days. In the figure K represents control group- pure hydrogel, B for BMP-2 loaded-hydrogel, V for VEGF-165 loaded-hydrogel and BV for hydrogel loaded with both BMP-2 and VEGF-165.

Hydrogel loaded with BMP-2, VEGF-165 and both the cytokines were immersed in PBS to test the release behavior of these growth factors with the pure hydrogel as control. It is shown in Fig. 3A that groups of BMP-2 loading and both BMP-2 and VEGF-165 loading had a burst release of BMP-2 in the first 3 days. After that they both showed a sustained release for more than 3 weeks. The situation of VEGF-165 release was quite similar (Fig. 3B). It is interesting that in the group of BMP-2 and VEGF-165 loaded hydrogel, although the concentration of each cytokine was half as group BMP-2 or VEGF-165, the release trend was close to the one cytokine loaded system.

### Osteogenic differentiation of MC3T3 cells cultured on the growth factor-loaded hydrogel

**Figure 4:**
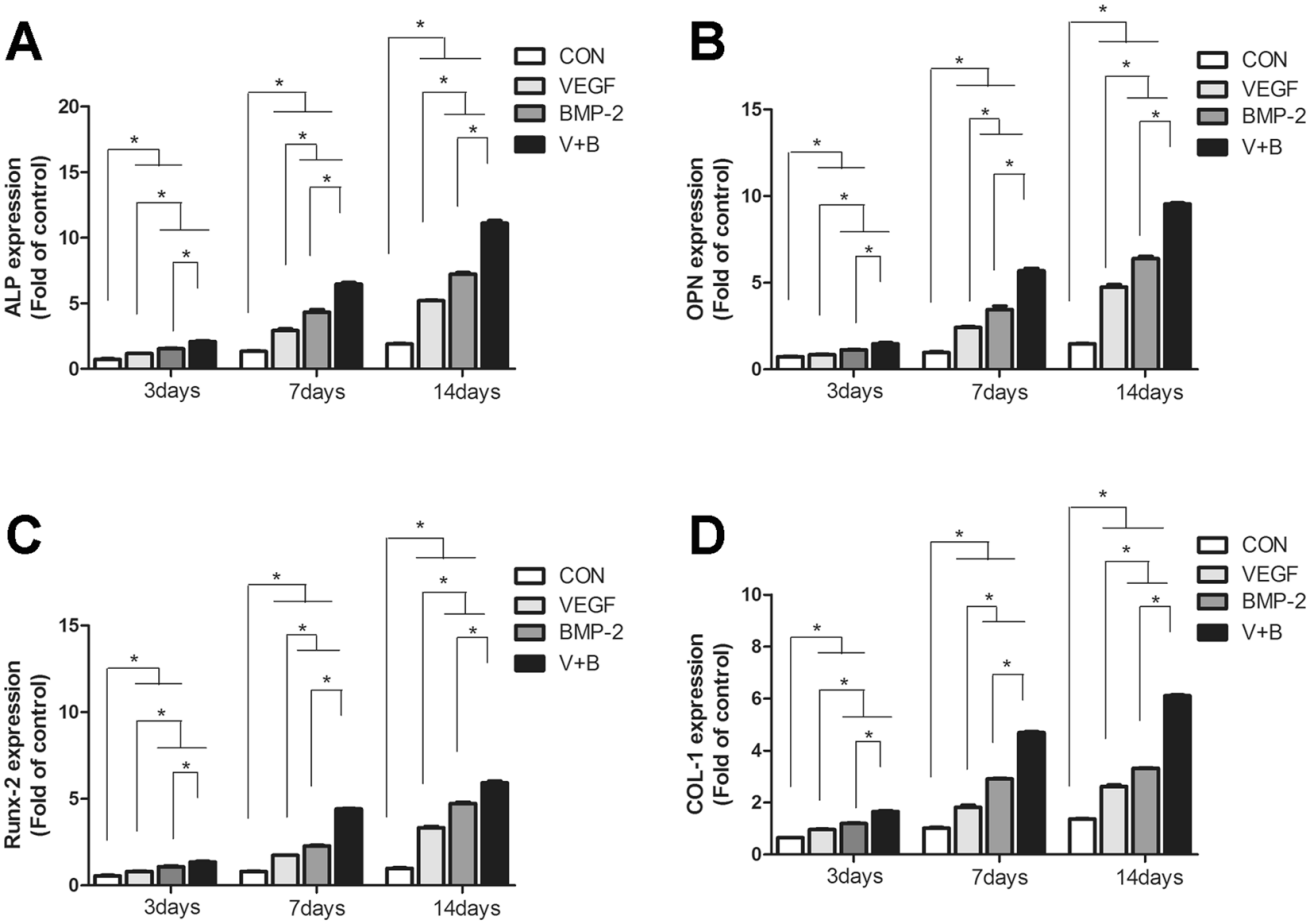
The ALP (**A**), COL I (**B**), Runx-2 (**C**) and OPN (**D**) gene expression of MC3T3 cells cultured on hydrogels of control, V, B and VB groups.

**Figure 5:**
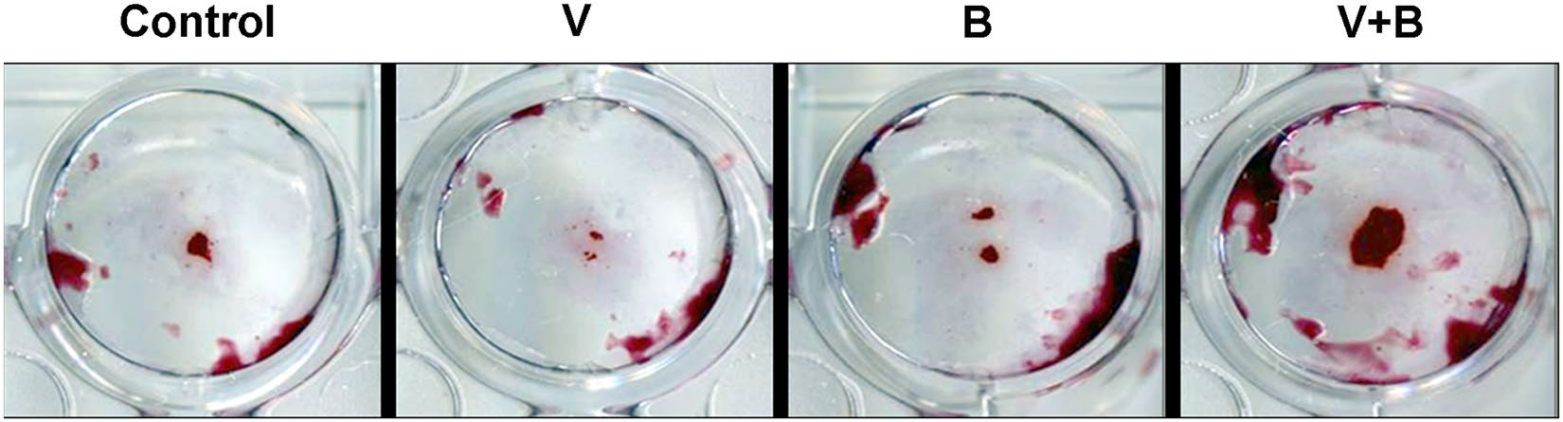
The formation of calcium nodule of the four groups by Alizarin Red S Staining.

Figure 4 showed the osteogenic gene expression of ALP, COL I, Runx-2 and OPN of MC3T3 cells of group V, B, VB and control. It is clearly revealed that the existence of both the cytokines (VB group) had best effect on the gene expression, significantly higher than other three groups. It is possibly resulted from a synergetic effect of BMP and VEGF comparing with the one-cytokine groups. Group B also showed a better impact on all the four gene expressions than group V, indicating that BMP-2 delivered a stronger improvement in osteogenic differentiation than that of VEGF. Alizarin Red S Staining analysis (Fig. 5) showed a consistent result in mineralization level, further proved the effect of cytokine release.

### Hydrogel degradation and subcutaneous ectopic ossification ***in vivo***

**Figure 6:**
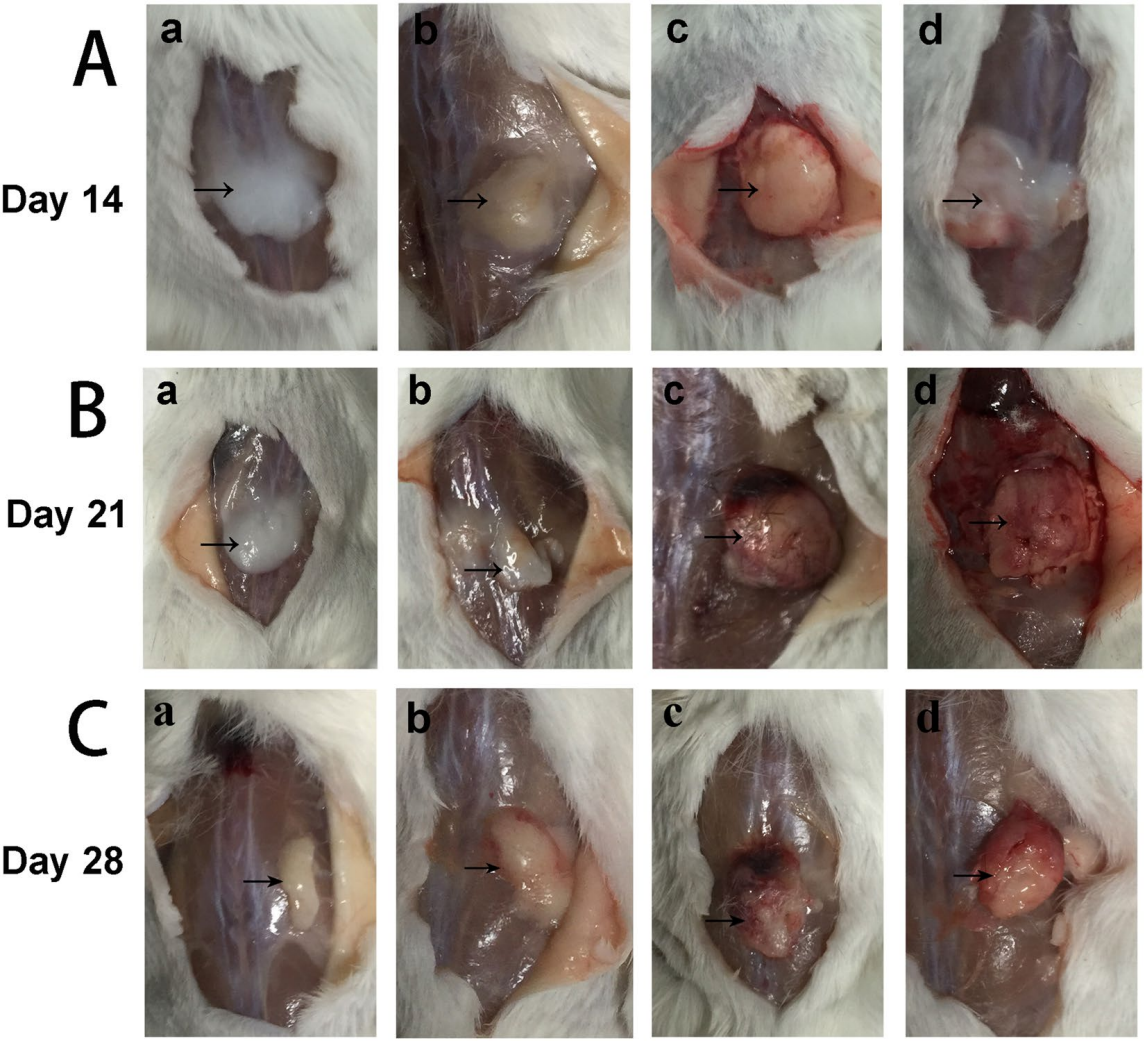
Hydrogel degradation under subcutaneous layer for 2 weeks (Line A), 3 weeks (Line B) and 4 weeks (Line C). lowercase letter a, b, c, d represent control group, V, B and VB group, respectively. Formation of bone-like hard tissue can be observed in B and VB group.

Injection of cytokine-loaded hydrogel (Control, V, B and VB) under subcutaneous layer was performed and then observed at 2, 3 and 4 weeks post-administration. The shape of hydrogel from control group and group V underwent a continuous shrink, became softer and no obvious ectopic ossification was observed although there were vascularization at the implanted site in group V, which was induced by the released VEGF from the hydrogel (Fig. 6b). Both group B (Fig. 6c) and VB (Fig. 6d) showed an increasing trend of the hydrogel area as well as the emerging of blood capillary and hard tissue in 2 weeks while that of group B was superior to VB. And the group VB exhibited a more significant formation of bone-like tissue than that of group B after 2 weeks.

**Figure 7:**
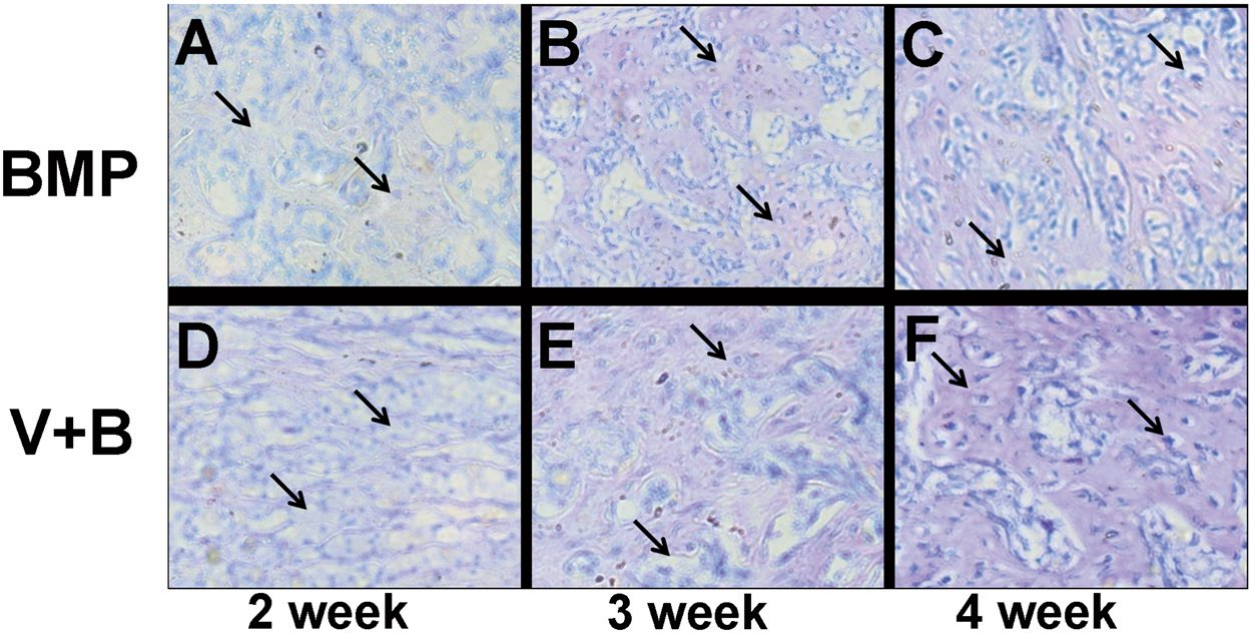
Toluidine blue staining of bone-like tissue B and BV group further proved the bone-like tissue formation.(**A**–**C**) are analysis after injection of hydrogel for 2, 3 and 4 weeks for (**B**) group and (**D**–**F**) are that of 2, 3 and 4 weeks for BV group, respectively.

Further analysis by Toluidine Blue Staining of hard tissue from group B and VB proved the newly-formed bone-like tissue (Fig. 7). It also indicated the increasing area from 2 to 4 weeks while the area of group VB was larger than that of B in all the 4 weeks.

### ***In vivo*** bone defect repair of hydrogel-loaded artificial bone scaffolds

**Figure 8:**
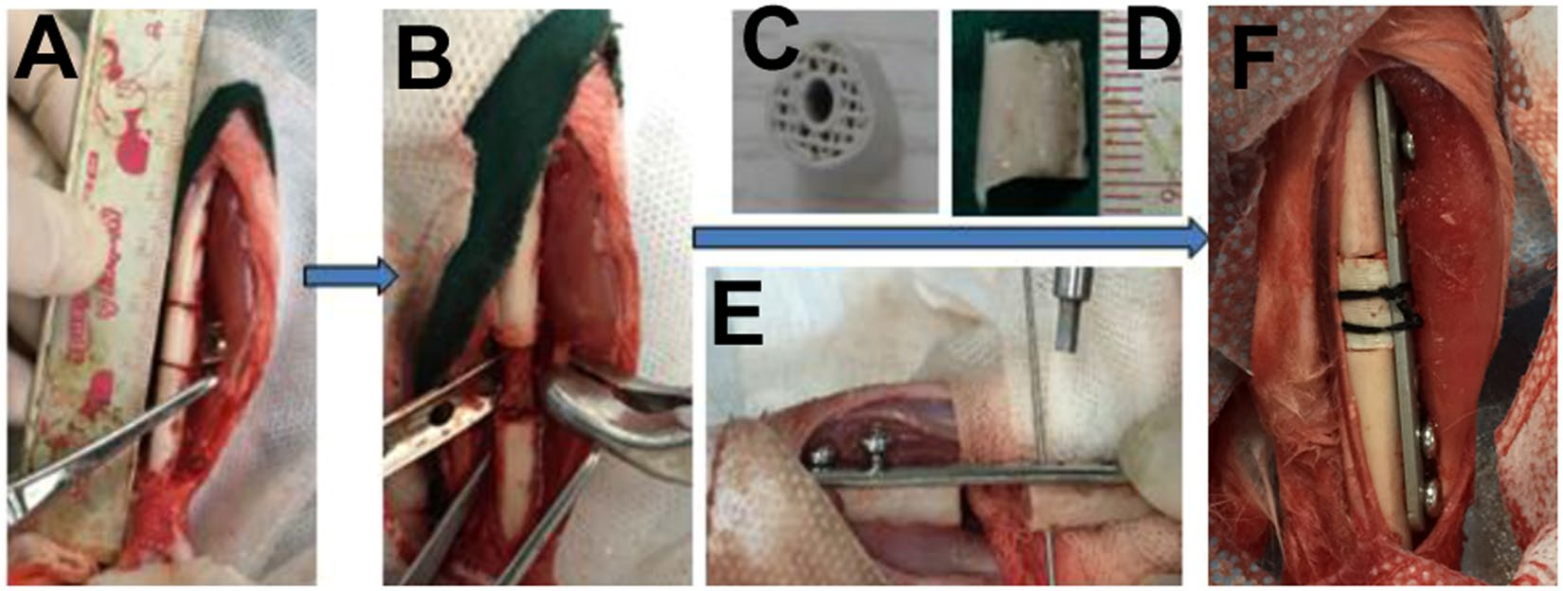
The operation process of aritificial bone scaffold in rabbit tibial defect. (**A**) exposure of surgical field, (**B**) construction of bone defect (1.2 cm), (**C**)anatomical biomimetic artificial bone scaffold, (**D**) osteotomy of autogenous bone, (**E**) fixation of miniplate and screw fixation system, (**F**) successful bone graft transplant into bone defect.

**Figure 9:**
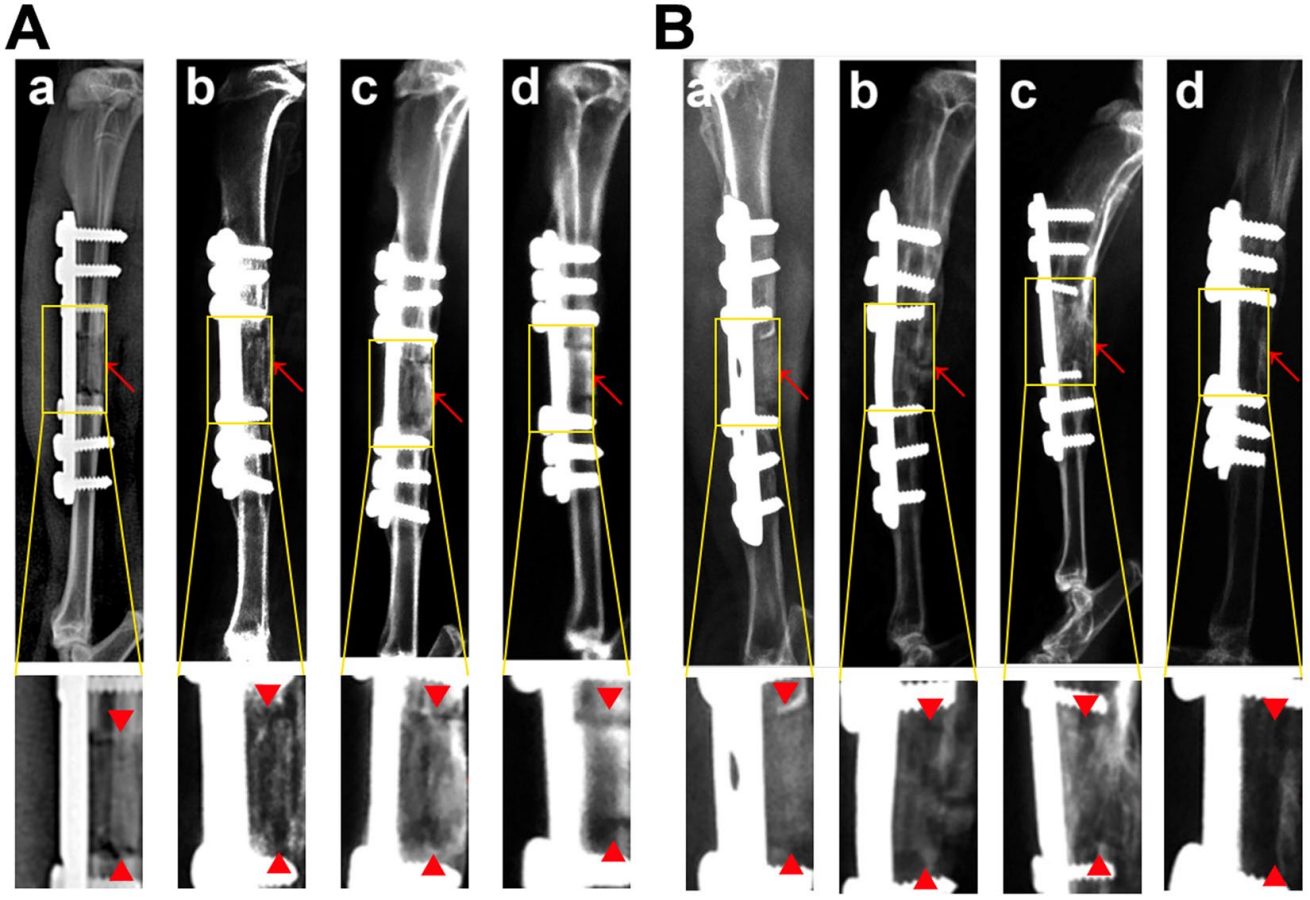
X-ray scanning of control (a), B (b), VB (c) groups and autogenous bone (d) after implantation for 4 weeks (**A**) and 12 weeks (**B**). The lower images were magnified from the corresponding regions. Red arrows indicate the bone defect edges.

**Figure 10:**
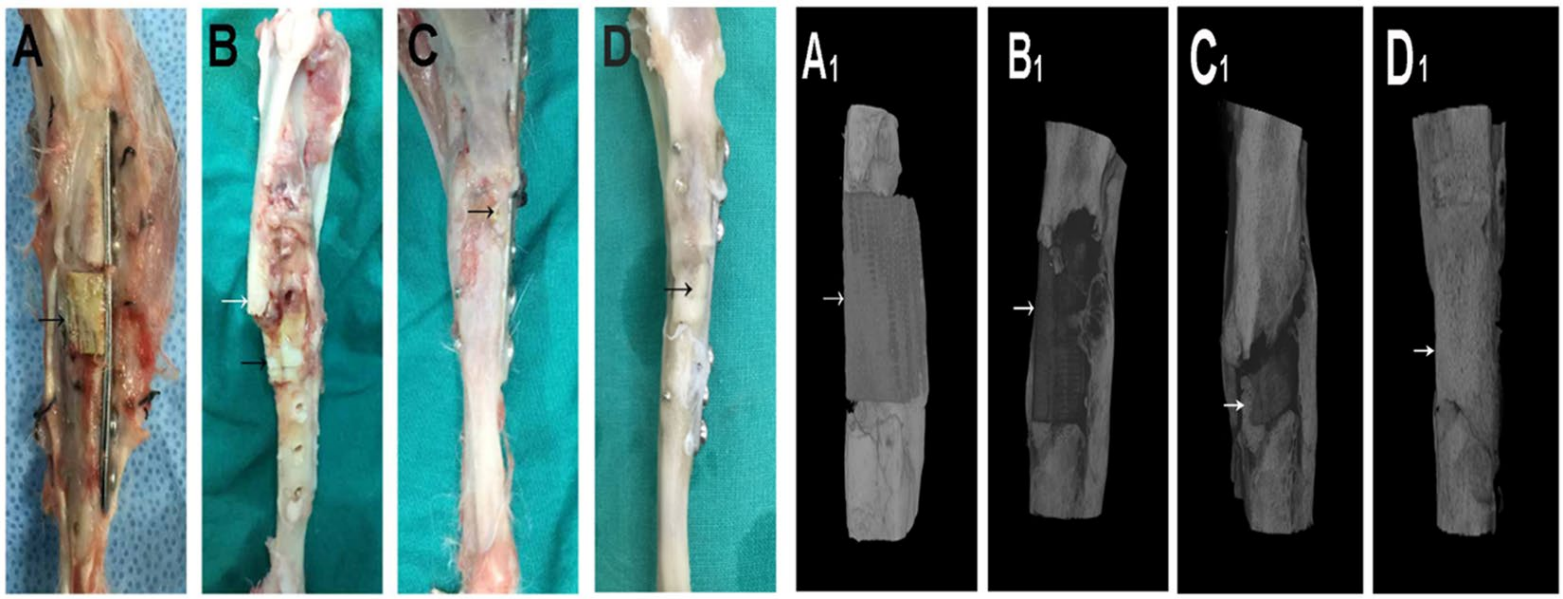
Gross appearance and CT reconstruction images of control (**A**,**A**_**1**_), B (**B**,**B**_**1**_), BV (**C**,**C**_**1**_) groups and autogenous bone (**D**,**D**_**1**_) for 12 weeks, arrows indicate the bone graft.

**Figure 11:**
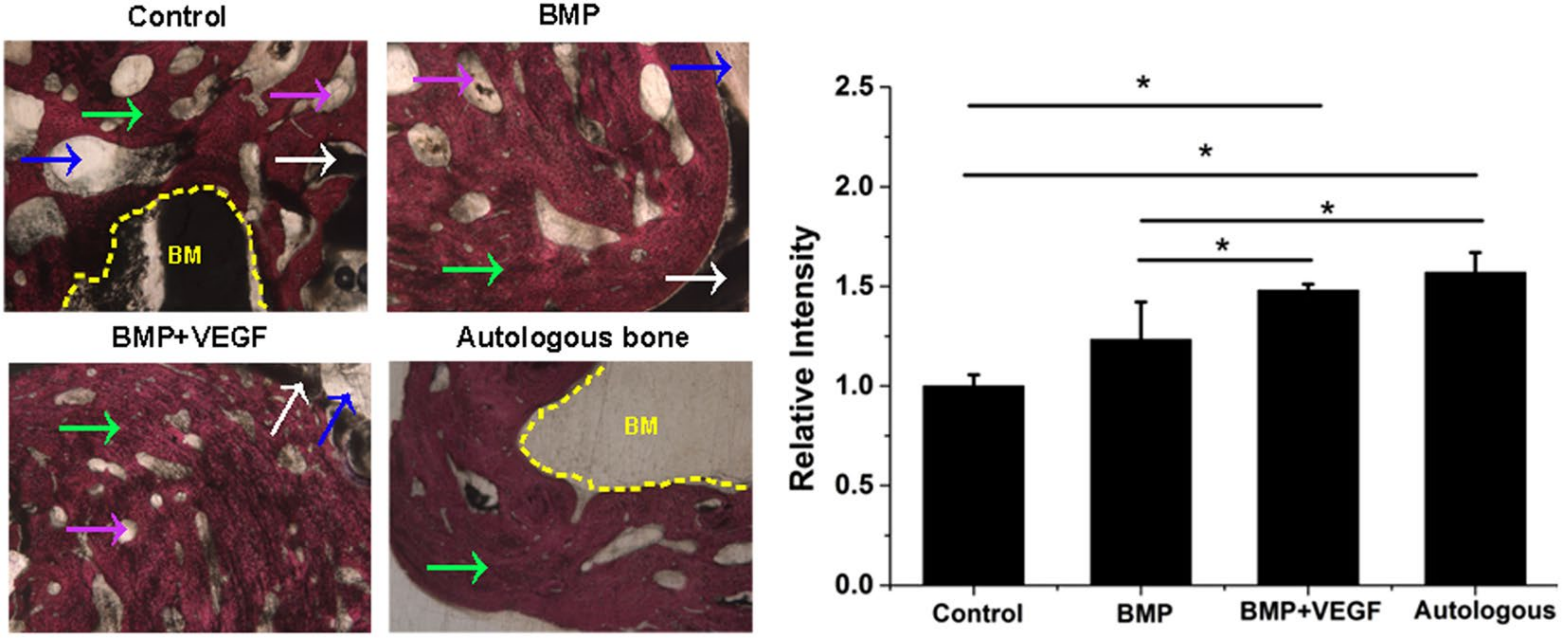
The VG staining of newly formed bone on the interface of implanted scaffolds/autogenous bone and nature bone at defect site of four groups (40×) with relevant statistics analysis. Pink arrow refers to PCL pieces, White arrow refers to HA pieces, Blue arrow refers to artificial scaffold,green arrow refers to new bone (NB), yellow area refers to bone marrow cavity (BM).

**Figure 12:**
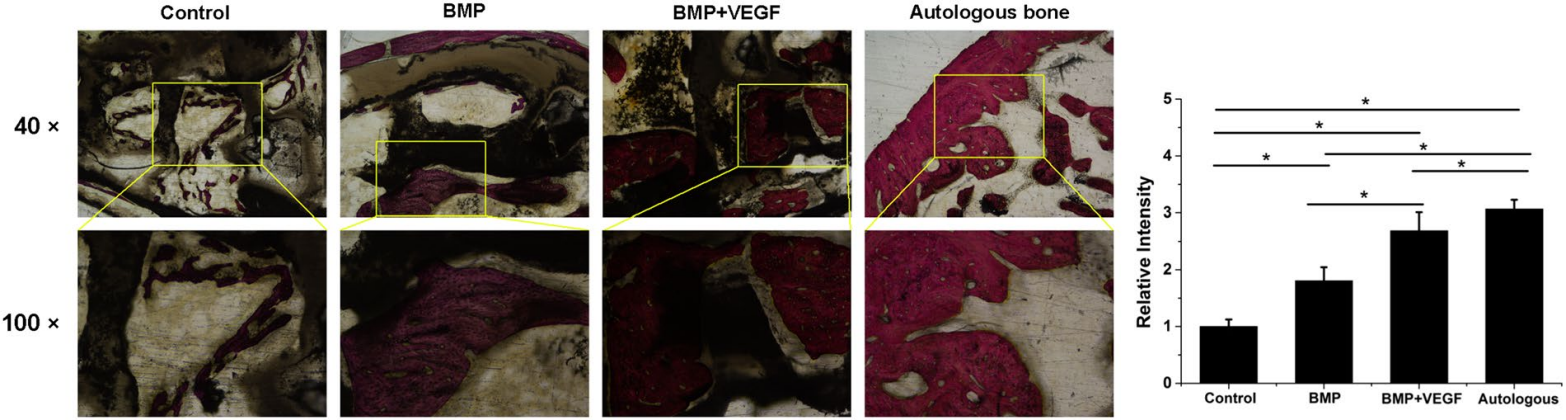
The VG staining of newly formed bone in the middle part inside the implanted scaffolds/autogenous bone of the four groups with relevant statistics analysis. The lower images were magnified from the corresponding regions.

A 1.2 cm-long tibial defect was constructed and scaffolds with pure hydrogel, group B and group VB hydrogel were planted into the defect site. The autogenous bone was used as positive control (Fig. 8A–F). The artificial bone scaffolds/bone were fixed and the rabbits were all alive during the experimental period. After 4 weeks of implantation, the gap and fracture zones were clearly seen in the autogenous bone group but there was no gap shown in the artificial bone scaffolds of other three groups. Especially, in B and VB group the fracture line became fuzzy, followed by the disappearance of osteotomy gap (Fig. 9A). However, the gap and fracture zones of autogenous bone group disappeared and new bone could be clearly seen at the defect sites after 12 weeks, which have similar repair effects to the B and VB group after 12 weeks, while the boundary line remained in pure hydrogel-scaffold group (Fig. 9B). A similar result was shown by the gross appearance in Fig. 10. Further analysis by CT reconstruction revealed that the scaffold of VB group was almost covered by the newly-formed callus at the defect site and they were connected so tightly that the fracture line could hardly be found, indicating favorable bone-implant osseointegration (Fig. 10C_1_). It is obvious that both the B and VB group showed better regeneration than the pure hydrogel-scaffold group, of which the implanted scaffold was still not osseointegrated with the host tissue (Fig. 10A_1_). And in the positive control group of autogenous bone, the defect was perfectly repaired without isolating from the host tissue (Fig. 10D_1_). The analysis by Van Gieson staining of new bone-like tissue at the interfaces between implanted materials and the surrounding bones (Fig. 11), as well as the intermediate part of the implants showed a consistent result (Fig. 12). It is obvious that with the addition of both BMP-2 and VEGF-165 in the system, the scaffold could obtain the best repair of defect.

## Discussion

Biomimetic bone scaffolds for the segmental defect in weight-bearing bone repair have been studied for years. However, the weak biomechanics, unmatched bone-scaffold interface and insufficient internal bone formation inhibited their further application in clinical use^24, 25^. In this study, the 3D printed HA/PCL biomimetic artificial bone scaffolds were fabricated individually according to the animal defect model and mimiced both the chemical composition (inorganic/organic materials) and the hierarchical structure (cortical bone/cancellous bone/medullary canal) of natural bones. Additionally, with the spatiotemporally controlled release of functional cytokines (VEGF-165, BMP-2) from the thermosensitive hydrogel which was adhered in the mimetic cancellous bone and medullary canal, the scaffolds can serve as an “anatomy-structure-function” trinity system, which showed favorable osteogenesis and angiogenesis capacity to avoid the currently used supraphysiologic doses and the concomitant adverse effects^26^. The hydrogel-embedded biomimetic scaffolds were used as an *in situ* carrier for BMP-2 or/and VEGF-165 delivery and was proved to be an effective way to achieve sustained release profile and meet the pathophysiological procedure of the nature bone fracture. The two cytokines of VEGF-165 and BMP-2 can produce synergetic effects on new bone formation at a reduced dosage. As indicated by the *in vivo* defect repair inspection, the trinity system could significantly promote bone repair at early stage and bone graft osseointegration later, showing its potential as effective substitute for bone autograft.

Numerous studies have been reported on the application of BMP-2 in various animal models so far^27, 28^, but there is a lack of consistency criterion on its optimum dosage for effective bone defect repair. Generally, the theraupetic dose of BMP-2 increases with the complexity of the developmental systems. The minimum dose of BMP-2 necessary to induce consistent bone formation is substantially higher in nonhuman primates than in rodents. For example, in rats 0.2–0.4 mg/mL of BMP-2 is effectively osteoinductive, whereas sheep and primates require higher concentrations of 0.43 and 0.75–1.5 mg/ mL of BMP-2^29–32^. Additionally, that BMP-2 above 0.15 mg/mL could develop side effects such as the fatty cyst in rat femoral segmental defect model has been reported^33^, let alone the toxic side effect of bone cyst, adipogenesis caused by BMP-2 of concentration 1.5 mg/mL used in clinical^26, 34^. In this study, the applied BMP-2 for rabbit model was set at 0.36 mg/mL or 90 µg in total between the concentration of rats and primates. As to the concentration of VEGF, it has been reported that the favorable synergistic effects of VEGF and BMP-2 depended on the specific ratio of these two cytokines. Inappropriate proportion could result in negative effect on related animal models of critical-sized defect and ectopic osteogenesis. For instance, the BMP-2 and VEGF ratio of 1/4 showed inhibition on bone formation while the ratio of 5/4 and above showed promotion^35–37^. Accordingly, the concentration of VEGF was set at a ratio of 1/10 that of BMP-2, to keep the VEGF concentration in a safe range. And half of the concentration of these two cytokines from V and B group was combined in group VB to study the synergistic effect.

As revealed in Fig. 3, the release of these two cykotines showed same behavior of a mild burst release at the third day and then a sustained slow release more than 21 days. The release profile of BMP-2 or VEGF is close to the zero-order release pattern due to the favourable degradation rate of the PLGA-PEG-PLGA copolymer. After cultured on the cytokine-loaded hydrogel, the osteogenic gene expression (ALP, COLI, RUNX2, OPN) of osteoblasts has revealed that both the VEGF-165 and BMP-2 showed stimulatory effects. ALP is a typical early markers for osteoblastic differentiation and Col I is a major matrix component of periodontal tissues as well as an early marker with the formation of extracellular matrix (ECM). Runx2 and OPN are master regulators in the commitment of osteogenesis. While BMP-2 indicates a more postive impact than that of VEGF-165 with the set concentration, it is interesting to notice that a combination of these two cytokines did show a significantly improvement. This was also proved by the results of calcium node staining. This may indicate that a sustained long-term and low dosage of cytokine supply, rather than a temporary burst release, is essential for the stimulatory property^38^.

The cytokine-loaded hydrogel was then subcutaneous injected in mice, with the pure hydrogel as control. The gel was formed *in situ* and the heterotopic ossification occurred after 7 days in B and VB group. At day 14, elliptical bone-like structures encysting hydrops was clearly observed in B group, while in VB group less but homogenous osteogenesis without cyst cavity was displayed. One of the possible reason is that higher dosage of BMP-2 can result in the side effects, which had occurred in previous reports of BMP-2 delivered by collagen sponges systems that caused encapsulated hematomas in new bone^39^. And another factor is the synergistic effect of the co-existed VEGF in VB group as VEGF has been proved to promote osteogenesis in the advanced stage^40^. Further analysis was conducted by toluidine blue staining (Fig. 7). A growing trend of new bone tissues were observed in both B and VB group in 28 days after the injection. Similarly in the aspect of angiogenesis, both B and VB group showed obvious neovascularization on the surface of the injected hydrogel and only a few vessels can be seen of the V group. For years, contradictory results have been reported on the effect of single high concentration of VEGF generating heterotopic ossification^41^. Our study here demonstrated that high dose of VEGF may lead to inhibition on the angiogenesis while the combination of VEGF and BMP-2 at relative low concentration in VB group possessed stimulatory property. The hydrogel matrix provided the system with favourable entrapment of cytokines and its desirable degradation rate *in vivo* result in a proper release rate. It mimiced the pathophysiological expression procedure of cytokines *in vivo*, which usually showed a sustained release of these cytokines in 5–21 days after bone fracture^42^, indicating the cytokine-loaded hydrogel has the potential to serve as the red bone marrow of natural bone. Compared to the far surpassed physiological doses to remedy rapid degradation and deactivation in body, a much lower concentration of the expensive cytokines used in the thermogel matrix could reduce the cost and maintain the efficiency as well^38^. The HA/PCL 3D artificial bones (Fig. 1C) with both cancellous bonelike features (porous scaffold) and cortical bonelike features by CT-guided FDM not only possess the interconnected pores that have the potential to guide cell attachment and growth/ingrowth as several previously studied bioceramics^43^ and bioceramic/polymeric composite scaffolds^44^, but also have advantages as easy production for manufacturers, easy operability for surgeons, and patient satisfaction^45, 46^.

The cytokine-loaded hydrogel could fill into the pore spaces of the scaffold at room temperature just like bone marrow distributed in autogenous bone, which make the VEGF and BMP spatially and temporally controlled release at temperature (37 °C) to avoid the currently used supraphysiologic dosage and the concomitant adverse effects^47^. The implantation of artificial bone to segmental bone defect model was facile and quick (approximately 5 min), following by direct screwing of a steel plate across it precisely, indicating that the mechanical strength and structure property of HA/PCL is suitable in the clinical trials of load-bearing bone defects.

To the best of our knowledge, there is few report about construction of single defect model of weight-bearing bone of rabbit tibia, although rabbit is one of the most commonly used animals for medical research, approximately 35% of musculoskeletal studies^48^. The relative small size of the model makes it difficult for the assessment of the defect repair effect of multiple implant materials in weight-bearing bones, inhibiting its insert and fixation placement^49^. However, we succeed in constructing the defect model of single weight-bearing bone of rabbit tibia under conditions of tailor-made anatomized armor plate based on the knowledge of the anatomy of the surgical region and skilled surgical technique. Additionally, during the whole implantation process and the subsequent 3 months, the rabbits in this study did not show obvious discomfort, and no dislocation and deformity in the *in situ* restoration of tibial defect, which testify that the experimental model of completely segemental bone defect repair and reconstruction is stable and indicate that the HA/PCL artificial bones could potentially promote high patient satisfaction^50, 51^.

After 4 weeks of implantation, the gap and fracture zones was clearly seen in the autogenous bone group but there was no gap shown in the artificial bone scaffolds of other three groups. Scaffolds fabricated by CT-guided FDM techinque can exactly match the original defect by osteotomy compared to the inevitable damage of autogenous bone and various autogenous bone of other parts, preventing the efficient repair and possible failure by the large gap. The fracture line became fuzzy, followed by the disappearance of osteotomy gap in B and VB group, indicating that besides the good bioactivity of HA/PCL composite scaffolds, the cytokines released from hydrogel could significantly promote bone repair in early stage. After 12 weeks of implantation, defects were repaired well in all the autogenous bone, B and VB groups as no fracture line was observed in X-ray scans. Specifically, the VB group showed evenly wrapped callus while that of B group showed uneven callus (pointed out by arrows in Fig. 9B-b). This may be caused by the lack of VEGF of B group and failure to induce angiogenesis to transmit signals from the internal gel when the scaffold surface underwent mineralization^52, 53^. And it is reported that a single high BMP-2 dose usually dysregulates Wnt signaling pathway and activate PPARg to promote adipogenesis over osteoblastogenesis and generate poor quality of new bone^53^. Consistent results were shown by the gross appearance, CT reconstruction images (Fig. 10) and histology detection at the interfaces and the intermediate part of the implants (Figs 11 and 12). The HA/PCL artificial bone equipped with sustained cytokine release hydrogel system, successfully mimic the structure and function of autogenous bone and showed potential substitutes of autogenous bone in large weight-bearing bone defect repair.

In conclusion, a series of studies from the individually 3D printed artificial bone using HA/PCL composite with CT reconstruction, the stability and release profile of cytokine-loaded hydrogel and effects on the biological characteristics of osteoblasts, to the ectopic ossification and repair of bone defect in the original site *in vivo* has been conducted. The results has illustrated that the dual-cytokine loaded hydrogel can play a role of red bone marrow of natural bone to promote osteogenesis. Its successful combination into scaffolds could further build a functional artificial bone which mimiced the autogenous bone with more precisely match to defect site. The “structure-anatomy-function” trinity strategy for large weight-bearing bone regeneration can promote osteogenesis and accelerate bone repair with less costly cytokines requirement. It is also implied that with proper design of materials, suitable cytokine induction and individual fabrication technique of artificial bone, efficient bone tissue repair could be achieved without preimplantation cell seeding.

## Methods

### Fabrication and characterization of artificial bone scaffolds

Nano-hydroxyapatite (HA) powder and polycaprolactone (PCL,Mw 60000) were purchased from Sigma Ltd. USA. To prepare the materials for 3D fabrication, HA powder was mixed with PCL at a mass ratio of 30/70 and stirred throughly (speed of 100 rpm/min) at 100 °C for 10 min using a twin counter-rotating internal mixer. The 3D model was reconstructed by CT based on the data collected from the scanning process of a normal New Zealand white rabbit leg. Then Mimics software (Ver.9.1, USA) were applied to segment the images and isolate the bony structure (Fig. 2A,B). After a typical long (1.2 cm) weight-bearing tibia model that mimics cortical and cancellous structure was obtained,the prepared HA/PCL paste was used to fabricate the artificial bone scaffold by a Fused Deposition Modeling (FDM) machine. The gross appearance and internal structure of scaffolds were obtained by photograph and CT scanning, respectively.

The compressive strength of the as-prepared scaffolds was measured along the longitudinal axis using a universal testing machine (Zwick Z005) at a loading rate of 5mm/min^54^. The tensile modulus was calculated according to the initial slope of the stress-strain curves^55^. Autogenous bone from the animal model and commercial BAM artificial bones (synthetic compoud of calcium sulfate salts) were selected as control groups. Six samples were measured for each group.

### Preparation and characterization of PLGA-PEG-PLGA hydrogel

Polyethylene glycol (PEG,Mw 1500, Sigma-Aldrich), D,L-lactide (LA, Purac), glycolide (GA, Purac) and stannous octoate (Sigma-Aldrich) were used to synthesize PLGA-PEG-PLGA triblock copolymers through the bulk ring-opening copolymerization process. The composition and structure of the copolymer were tested by^1^H-NMR and data were recorded with a Bruck DMX500 spectrometer operating at 500 MHz using CDCl_3_ as a solvent and tetramethylsilane (TMS) as an internal standard. The molecular weight and its distribution of PLGA-PEG-PLGA were examined by Gel permeation chromatography (GPC, Agilent 1100). Tetrahydrofuran (THF) was used as the mobile phase at a flow rate of 1 mL/min at the temperature of 22–25 °C. The columns were calibrated with polystyrene (PS) standards.

The temperature-induced sol-gel transition of PLGA-PEG-PLGA triblock copolymer in saline was determined using the vial inversion approach. 25 wt% copolymer-saline solution was prepared in vials and continuously stirred in a water bath with controlled temperature increasing. The criterion of a gel was defined if no visual flow was observed within 30 s after inversion of the vial. Rheological analysis (Mavlern Kinexus) was conducted to measure the sol-gel transition. The heating rate was 0.5 °C/min starting from 15 °C and the angular frequency was set at 10 rad/s.

### The growth factors loading and release behaviour in PLGA-PEG-PLGA hydrogel

40 mL of 25 wt% copolymer-saline solution was disinfected with irradiation (7 kGy) and kept at 4 °C for 24 h to maintain homogeneous. Then 14 mL of the hydrogel was injected into the reagent bottle containing 500 μg rhVEGF-165 and 5 mg rhBMP-2 at room temperature in a clean bench and mixed thoroughly, marked as group V and group B, respectively. Same volume of solution from the V and B group was mixed to obtain the hydrogel loaded with both rhVEGF-165 and rhBMP-2 (group VB). The pure hydrogel was used as control. All the samples were kept at 4 °C for the following experiments.

To analyse the release of growth factors from the hydrogel, PBS (containing 1% penicillin-streptomycin, PS) was selected as buffer solution. 0.25 mL of the above hydrogels was added to the 48-well plate and maintained at 37 °C incubator for 10 min to obtain gel in each well. After that 1000 μL of PBS was added. The plate was kept in 37 °C incubator during the process. At time point 24 h, 3 d, 7 d, 14 d and 21 d, the supernatant was collected and fresh PBS was added. The concentraion of rhBMP- 2/rhVEGF-165 was measured by ELISA Kit according to the manufacturer’s instruction.

### Osteogenic differentiation of MC3T3 cells cultured on the growth factor-loaded hydrogel

MC3T3 cells were cultured in Dulbecco’s Modified Eagle Medium (DMEM) supplemented with 10% fetal bovine serum (FBS) and 1% PS in a humidified CO_2_ incubator at 37 °C. 0.5 mL of V, B, VB and pure hydrogel samples for each well was added to 6-well plate and maintaind for 5 min in a incubator to obtain gel. After that MC3TC cells were seeded at a density of 10^5^ cells per well for 3 d, 7 d and 14 d with the DMEM refreshed every two days. At each time point, the culture medium was removed and the cells were washed three times with PBS, followed by addition of TRIzol reagent to isolate the total RNA. Complementary DNA was synthesized from 1 μg of total RNA using DyNAmo^TM^ cDNA Synthesis Kit following the manufacture’s protocol(RNA primer were shown in Table 2). SYBR Green qPCR Master Mix was used for detection and the target mRNA expressions were assayed on the ABI Prism 7300 Thermal Cycler. Each sample was performed in triplicate. The mean cycle threshold (Ct) value of each target gene was normalized against Ct value of a house keep in gene β-actin. The ΔΔCt method was applied to compare the mRNA expression of different groups.

**Table 2:** The primer sequences of qRT-PCR.

Genes	Forward primer	Reverse Primer
β-actin	TGTTACCAACTGGGACGACGACA	CTGGGTCATCTTTTCACGGT
ALP	TGACCTTCTCTCCTCCATCC	CTTCCTGGGAGTCTCATCCT
COL1	CAGGGTATTGCTGGACAACGTG	GGACCTTGTTTGCCAGGTTCA
RUNX2	TCCTGTAGATCCGAGCACCA	CTGCTGCTGTTGTTGCTGTT
OPN	ACTTTTCTCGTTTGTGGAGC	GAACCCAGGTGTCTCCAAGA

Alizarin Red S staining was used to highlight mineralization nodules in MC3TC cultured with the hydrogels. After washed with PBS, the remained cells were fixed in 4% paraformaldehyde for 20 minutes followed by 3 times washing with PBS. Then the cells were stained in a solution of 1% Alizarin Red in 37 °C incubator for 20 minutes. The samples were air-dried and images acquired with a digital camera.

### Hydrogel degradation and subcutaneous ectopic ossification ***in vivo***

ICR mice of either gender, aged 8 weeks(22 ± 2 g) were selected as model animals to test the hydrogel degradation *in vivo*. In a typical process, the mice were anesthetized by diethyl ether. Then 0.25 mL of 25 wt% copolymer solution of control, V, B and VB groups was injected into the subcutaneous layer of each mouse with a 1 mL syringe. After injection for the predetermined time intervals (24 h, 3 d, 7 d, 14 d, 21 d and 28 d), the mice were sacrificed and the state of remaining gels as well as the subcutaneous ectopic ossification were observed. Toluidine blue staining of bone-like tissue of the experimental groups were applied to further analyse the ossification on the hydrogel-injected parts. All the tests were carried with 3 samples for each group. The animal experiments were approved by the Ethics Committee of Changzheng Hospital in accordance with Principles of Laboratory Animal Care.

### Preparation of hydrogel-loaded artificial bone scaffolds and the bone defect repair ***in vivo***

The as-prepared artifical bone scaffolds were immersed in 75% ethyl alcohol solution for 12 h and then washed with PBS for several times. Every 3 scaffolds were put into a 2 mL blood sedimentation tube, followed by the vacuumizing process with a 50 mL syringe repeatly. Then the rhVEGF-165/rhBMP-2 loaded hydrogel and pure hydrogel was injected to the tube via the rubber channel to immerse the scaffolds. The tubes were kept at 4 °C for 2 h to make the hydrogel loaded in the inner of scaffolds thoroughly.

6 month-old New Zealand white rabbits (3 kg) were divided into three groups randomly, shaved and anesthesized before the operation. A complete tibia bone defect model (1.2 cm) was employed in this work in accordance with the CT scanning results in 2.1. The defects were then reconstructed with the autogenous bone, pure hydrogel, rhBMP2-hydrogel and rhBMP2/rhVEGF165-hydrogel loaded-scaffolds prepared above. The experimental animals were sacrificed after 4 weeks and 12 weeks for observation and CT scanning. Samples from the implanted parts of 12 weeks were fixed in 10% formalin for 2 d and washed with distilled water for 4 h. The samples were dehydrated in grades ethanol solution (75%, 85%, 95%, 100%) for 2 d each. After embedded in the medium of poly(methyl methacrylate), the samples were sectioned with a microtome (Leica SP1600) and polished. Van Gieson staining (VG staining) was applied to observe the new bone formation of the implanted parts, the relevant statistic analysis was conducted via Image Pro software.

### Statistical analysis

**Figure 13:**
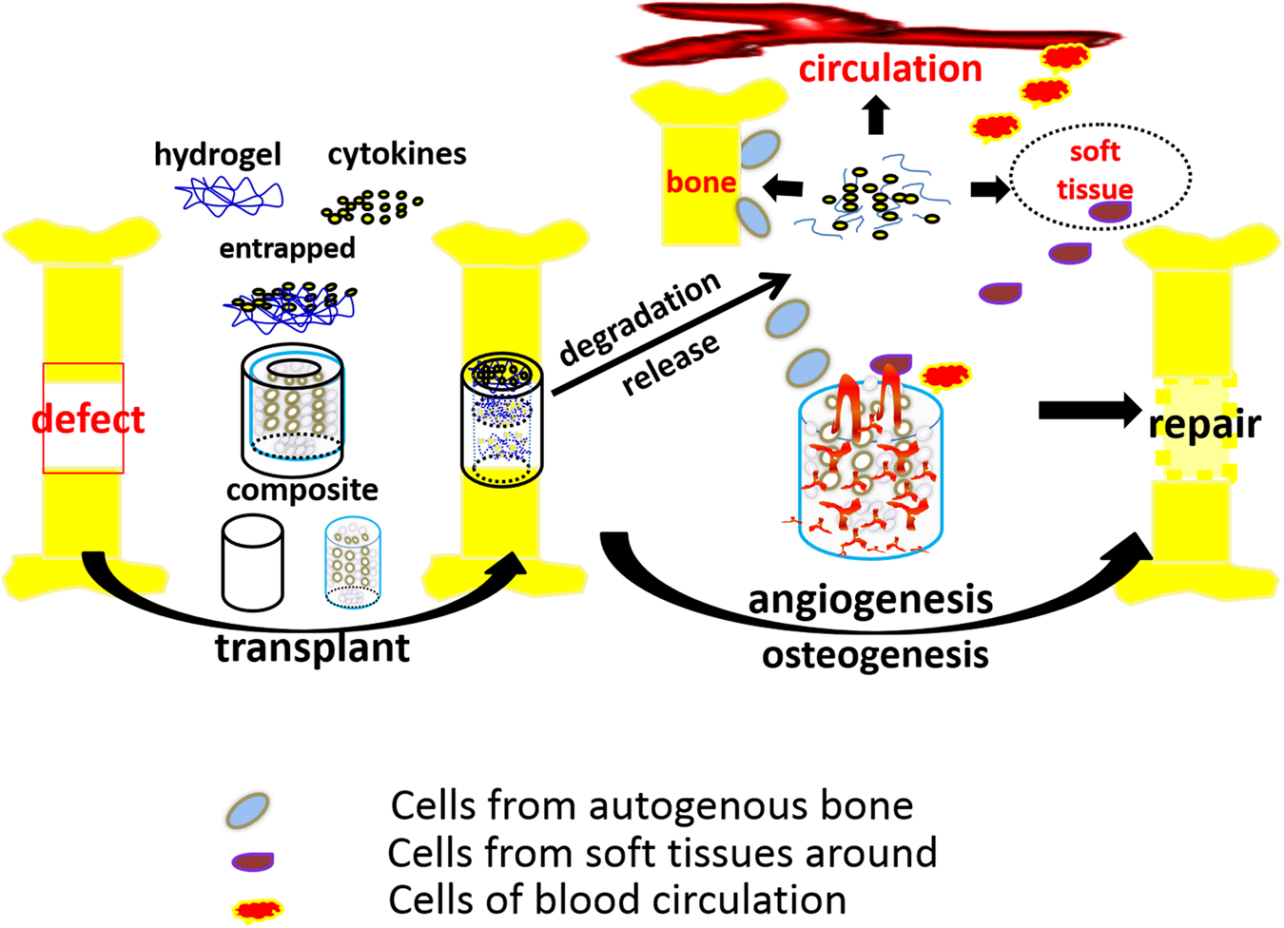
Cytokines-loaded hydrogel was combined with the CT-guided 3D-printing artificial bone scaffolds and implanted into the complete tibial defect (1.2 cm). With the mechanical support and biocompatibility of the porous scaffolds, the sustained release of cytokines and the stimulation on angiogenesis and osteogenesis, the defect was successfully repaired.

All the statistical computation was performed using SPSS software and the statistical significance was analysed using T-Test. All the data are shown as means ± standard deviation (SD) and the level of significance was set at *p* < 0.05 Fig. 13.

## References

[1] O’Keefe RJ, Mao J (2011). Bone tissue engineering and regeneration: from discovery to the clinic–an overview. Tissue Eng Part B Rev.

[2] Soucacos PN, Dailiana Z, Beris AE, Johnson EO (2006). Vascularised bone grafts for the management of non-union. Injury.

[3] Liu Y, Lim J, Teoh SH (2013). Review: development of clinically relevant scaffolds for vascularised bone tissue engineering. Biotechnol Adv.

[4] Fillingham Y, Jacobs J (2016). Bone grafts and their substitutes. Bone Joint J.

[5] Einhorn TA, Gerstenfeld LC (2015). Fracture healing: mechanisms and interventions. Nature Reviews. Rheumatology.

[6] Thavornyutikarn B, Chantarapanich N, Sitthiseripratip K, Thouas GA, Chen Q (2014). Bone tissue engineering scaffolding: computer-aided scaffolding techniques. Progress in Biomaterials.

[7] Polo-Corrales L, Latorre-Esteves M, Ramirez-Vick JE (2014). Scaffold design for bone regeneration. Journal of Nanoscience & Nanotechnology.

[8] Amini AR, Laurencin CT, Nukavarapu SP (2012). Bone tissue engineering: recent advances and challenges. Critical Reviews in Biomedical Engineering.

[9] Santos MI, Reis RL (2010). Vascularization in bone tissue engineering: physiology, current strategies, major hurdles and future challenges. Macromol Biosci.

[10] Kannan RY (2005). The roles of tissue engineering and vascularisation in the development of micro-vascular networks: a review. Biomaterials.

[11] Bianco P (2015). Stem cells and bone: a historical perspective. Bone.

[12] Martino MM, Briquez PS, Maruyama K, Hubbell JA (2015). Extracellular matrix-inspired growth factor delivery systems for bone regeneration. Adv Drug Deliv Rev.

[13] Blokhuis TJ, Calori GM, Schmidmaier G (2013). Autograft versus BMPs for the treatment of non-unions: what is the evidence?. Injury.

[14] De Biase P, Capanna R (2005). Clinical applications of BMPs. Injury.

[15] Bessa PC, Casal M, Reis RL (2008). Bone morphogenetic proteins in tissue engineering: the road from laboratory to clinic, part II (BMP delivery). J Tissue Eng Regen Med.

[16] Carmeliet P, Jain RK (2011). Molecular mechanisms and clinical applications of angiogenesis. Nature.

[17] Kim YH, Tabata Y (2015). Dual-controlled release system of drugs for bone regeneration. Adv Drug Deliv Rev.

[18] Belair DG, Le NN, Murphy WL (2014). Design of growth factor sequestering biomaterials. Chem Commun (Camb).

[19] Li K (2013). A long-acting formulation of a polypeptide drug exenatide in treatment of diabetes using an injectable block copolymer hydrogel. Biomaterials.

[20] Chen Y (2016). Controlled release of liraglutide using thermogelling polymers in treatment of diabetes. Sci Rep.

[21] Zhang L (2015). Sustained intravitreal delivery of dexamethasone using an injectable and biodegradable thermogel. Acta Biomater.

[22] Yu L, Chang G, Zhang H, Ding J (2006). Temperature-Induced Spontaneous Sol–Gel Transitions of Poly(D,L-lactic acid-co-glycolic acid)-b-Poly(ethylene glycol)-b-Poly(D,L-lactic acid-co-glycolic acid) Triblock Copolymers and Their End-Capped Derivatives in Water. J Polym Sci A Polym Chem.

[23] Luan JB (2017). Positional isomeric effects of coupling agents on the temperature-induced gelation of triblock copolymer aqueous solutions. Polym Chem.

[24] Amorosa LF (2013). Physiologic load-bearing characteristics of autografts, allografts, and polymer-based scaffolds in a critical sized segmental defect of long bone: an experimental study. Int J Nanomedicine.

[25] Horner EA (2010). Long bone defect models for tissue engineering applications: criteria for choice. Tissue Eng Part B Rev.

[26] Oryan A, Alidadi S, Moshiri A, Bigham-Sadegh A (2014). Bone morphogenetic proteins: a powerful osteoinductive compound with non-negligible side effects and limitations. Biofactors.

[27] Boerckel JD (2011). Effects of protein dose and delivery system on BMP-mediated bone regeneration. Biomaterials.

[28] Lo KW, Ulery BD, Ashe KM, Laurencin CT (2012). Studies of bone morphogenetic protein-based surgical repair. Adv Drug Deliv Rev.

[29] Agarwal R, Garcia AJ (2015). Biomaterial strategies for engineering implants for enhanced osseointegration and bone repair. Adv Drug Deliv Rev.

[30] Bayer EA, Gottardi R, Fedorchak MV, Little SR (2015). The scope and sequence of growth factor delivery for vascularized bone tissue regeneration. J Control Release.

[31] Proceedings from the Workshop on Science-Based Assessment: Accelerating Product Development of Combination Medical Devices, in: B.A. Scarborough (Ed.), Proceedings from the Workshop on Science-Based Assessment: Accelerating Product Development of Combination Medical Devices, Washington (DC) (2004). 25009880

[32] Devices, R.-A. InFUSE™ Bone Graft/LT-CAGE™ Lumbar Tapered Fusion Device - P000058. http://www.fda.gov/MedicalDevices/ProductsandMedicalProcedures/DeviceApprovalsandClearances/Recently-ApprovedDevices/ucm083423.htm (2013).

[33] Zara JN (2011). High doses of bone morphogenetic protein 2 induce structurally abnormal bone and inflammation *in vivo*. Tissue Eng Part A.

[34] Dimar JR (2009). Clinical and radiographic analysis of an optimized rhBMP-2 formulation as an autograft replacement in posterolateral lumbar spine arthrodesis. J Bone Joint Surg Am.

[35] Bai Y (2013). Localized delivery of growth factors for angiogenesis and bone formation in tissue engineering. Int Immunopharmacol.

[36] Chen FM, Zhang M, Wu ZF (2010). Toward delivery of multiple growth factors in tissue engineering. Biomaterials.

[37] Farokhi M (2016). Importance of dual delivery systems for bone tissue engineering. J Control Release.

[38] Poldervaart MT (2013). Sustained release of BMP-2 in bioprinted alginate for osteogenicity in mice and rats. PloS one.

[39] Rodgers SD (2013). Revision surgery after interbody fusion with rhBMP-2: a cautionary tale for spine surgeons. J Neurosurg Spine.

[40] Yang YQ (2012). The role of vascular endothelial growth factor in ossification. Int J Oral Sci.

[41] Mayr-Wohlfart U (2002). Vascular endothelial growth factor stimulates chemotactic migration of primary human osteoblasts. Bone.

[42] Tsiridis E, Upadhyay N, Giannoudis P (2007). Molecular aspects of fracture healing: which are the important molecules?. Injury.

[43] Gao C (2014). Current progress in bioactive ceramic scaffolds for bone repair and regeneration. Int J Mol Sci.

[44] Rai B (2010). Differences between *in vitro* viability and differentiation and *in vivo* bone-forming efficacy of human mesenchymal stem cells cultured on PCL-TCP scaffolds. Biomaterials.

[45] Xu N (2014). 3D artificial bones for bone repair prepared by computed tomography-guided fused deposition modeling for bone repair. ACS Appl Mater Interfaces.

[46] Park SA, Lee SH, Kim WD (2011). Fabrication of porous polycaprolactone/hydroxyapatite (PCL/HA) blend scaffolds using a 3D plotting system for bone tissue engineering. Bioprocess Biosyst Eng.

[47] Samorezov JE, Alsberg E (2015). Spatial regulation of controlled bioactive factor delivery for bone tissue engineering. Adv Drug Deliv Rev.

[48] Neyt JG, Buckwalter JA, Carroll NC (1998). Use of animal models in musculoskeletal research. Iowa Orthop J.

[49] Pearce AI (2007). Animal models for implant biomaterial research in bone: a review. Eur Cell Mater.

[50] Do AV, Khorsand B, Geary SM, Salem AK (2015). 3D Printing of Scaffolds for Tissue Regeneration Applications. Adv Healthc Mater.

[51] Yu H (2009). Improved tissue-engineered bone regeneration by endothelial cell mediated vascularization. Biomaterials.

[52] Koike N (2004). Tissue engineering: creation of long-lasting blood vessels. Nature.

[53] Krause U (2010). Pharmaceutical modulation of canonical Wnt signaling in multipotent stromal cells for improved osteoinductive therapy. Proc Natl Acad Sci USA.

[54] Sun G (2013). Novel biodegradable electrospun nanofibrous P(DLLA-CL) balloons for the treatment of vertebral compression fractures. Nanomedicine.

[55] Zhao J (2009). Improving mechanical and biological properties of macroporous HA scaffolds through composite coatings. Colloids Surf B Biointerfaces.

